# Molecular detection of parapoxvirus in Ixodidae ticks collected from cattle in Corsica, France

**DOI:** 10.1002/vms3.700

**Published:** 2022-01-28

**Authors:** Vincent Cicculli, Nazli Ayhan, Léa Luciani, Laura Pezzi, Apolline Maitre, Dorine Decarreaux, Xavier de Lamballerie, Jean‐Christophe Paoli, Laurence Vial, Remi Charrel, Alessandra Falchi

**Affiliations:** ^1^ Laboratoire de Virologie Université de Corse‐Inserm Corte France; ^2^ Unité Des Virus Emergents (UVE: Aix Marseille Université IRD 190, Inserm 1207, IHU Méditerranée Infection) Marseille France; ^3^ UR045 Laboratoire de Recherches sur le Développement de l’Élevage Institut National de la Recherche pour l'Agriculture l'Alimentation et l'Environnement Corte France; ^4^ UMR CIRAD‐INRA ASTRE (Animal, Health, Territories, Risks and Ecosystems) Department BIOS, Campus International de Baillarguet Montpellier France

**Keywords:** cattle, epidemiology, ticks, zoonoses

## Abstract

**Background:**

Several viruses belonging to the family Poxviridae can cause infections in humans and animals. In Corsica, livestock farming (sheep, goats, pigs, and cattle) is mainly mixed, leading to important interactions between livestock, wildlife, and human populations. This could facilitate the circulation of zoonotic diseases, and makes Corsica a good example for studies of tick‐borne diseases.

**Objectives:**

To gain understanding on the circulation of poxviruses in Corsica, we investigated their presence in tick species collected from cattle, sheep, horses, and wild boar, and characterized them through molecular techniques.

**Methods:**

Ticks were tested using specific primers targeting conserved regions of sequences corresponding to two genera: parapoxvirus and orthopoxvirus.

**Results:**

A total of 3555 ticks were collected from 1549 different animals (687 cattle, 538 horses, 106 sheep, and 218 wild boars). They were tested for the presence of parapoxvirus DNA on one hand and orthopoxvirus DNA on the other hand using Pangeneric real‐time TaqMan assays. Orthopoxvirus DNA was detected in none of the 3555 ticks. Parapoxvirus DNA was detected in 6.6% (36/544) of ticks collected from 23 cows from 20 farms. The remaining 3011 ticks collected from horses, wild boars, and sheep were negative. The infection rate in cow ticks was 8.0% (12/148) in 2018 and 6.0% (24/396) in 2019 (*p* = 0.57). Parapoxvirus DNA was detected in 8.5% (5/59) of Hyalomma scupense pools, 8.2% (15/183) of Hyalomma marginatum pools, and 6.7% (16/240) of Rhipicephalus bursa pools (*p* = 0.73). We successfully amplified and sequenced 19.4% (7/36) of the positive samples which all corresponded to pseudocowpox virus.

**Conclusions:**

Obviously, further studies are needed to investigate the zoonotic potential of pseudocowpox virus and its importance for animals and public health.

## Graphical abstract

Detection and characterization of poxvirus detected in ticks collected from wild and domestic animals in Corsica. Parapoxvirus DNA was detected in 6.6% (36/544) of ticks collected from 23 cows from 20 farms. All Corsican pseudocowpox viruses clustered with previously published European sequences of Pseudocowpox viruses detected in cattle and humans.

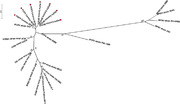

## 1 INTRODUCTION

Viruses belonging to the orthopoxvirus and parapoxvirus genera are large, enveloped, linear double‐stranded DNA viruses in the family Poxviridae (McFadden, 2005). Poxviruses are of major veterinary and human importance and infect various vertebrates and invertebrates, including humans. The genus *Parapoxvirus* contains five virus species: orf virus, bovine papular stomatitis virus, pseudocowpox virus, and parapoxvirus of red deer in New Zealand (Buttner & Rziha, 2002). There are three known zoonotic orthopoxvirus species: monkeypox virus, cowpox virus, and vaccinia virus which are associated with outbreaks in Africa, Europe, South America, and Asia (Singh et al., 2007). Humans are susceptible to monkeypox virus, cowpox virus, vaccinia virus, bovine popular stomatitis virus, orf virus, and pseudocowpox virus. Although the complete host range of these viruses is unclear, domestic animals such as sheep, goats, cats, dogs, and dairy cows can be infected with orthopoxvirus and/or parapoxvirus (Cicculli et al., 2020). Infected humans play an important role in the spread of orthopoxvirus and parapoxvirus among domestic animals, especially during milking and other livestock‐related occupational activities (Cicculli et al., 2020; McFadden, 2005). Clinically, the exanthematous lesions caused by zoonotic orthopoxvirus and parapoxvirus species are very similar, especially in humans and cows, and can be diagnosed in areas of orthopoxvirus/parapoxvirus cocirculation (Inoshima et al., 2000).

Recently, the presence of two parapoxvirus (pseudocowpox virus and bovine popular stomatitis virus) was reported in ticks collected from zebu cattle in Eastern Burkina Faso (Ouedraogo et al., 2020). Although the natural interaction between ticks and the detected parapoxvirus in that study is unknown, this finding shows that ticks may be a good indicator of the spread of these pathogens.

In Corsica, a French Mediterranean island, ticks of the genus *Ixodes, Hyalomma*, *Dermacentor*, *Haemaphysalis*, and *Rhipicephalus* have been identified and can act as vectors for a variety of emerging diseases (Cicculli, Capai, et al., 2019; Cicculli, de Lamballerie, et al., 2019; Cicculli et al., 2020; Cicculli, Masse, et al., 2019; Cicculli, Oscar, et al., 2019; Grech‐Angelini et al., 2020). Since mixed livestock farming (sheep, goats, pigs, and cattle) is extensive in Corsica, high interactions between livestock, wildlife, and human populations can facilitate the circulation of zoonotic diseases in the island. To our knowledge, there has been no investigation of the presence of poxviruses in domestic and wild animals in Corsica. Thus, the aim of this study was to provide new information about the potential circulation of parapoxvirus and orthopoxvirus by investigating their presence in tick species collected from cattle, sheep, horses, and wild boars in Corsica.

## 2 MATERIALS AND METHODS

### 2.1 Study area and collection of ticks

**FIGURE 1:**
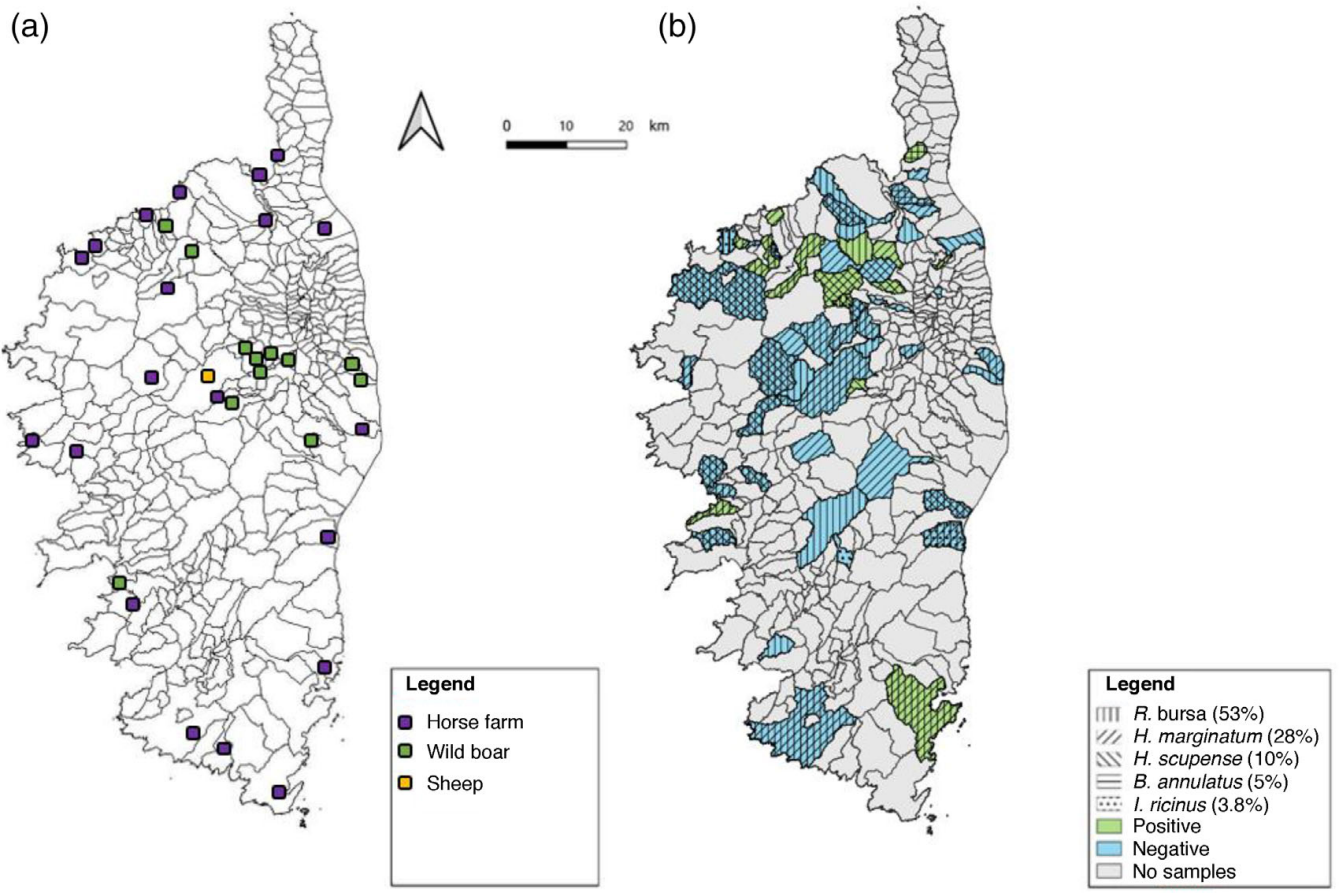
(a) Map of Corsica, France, indicating the tick collection sites and the animal species and farm and (b) tick species and positive pools of ticks collected from cattle in the study area, Corsica. *R. sanguineus* (*n* = 6) and *H. punctata* (*n* = 4) were not included

Ticks were collected (i) in May and June, 2019 from one sheep‐breeding farm located in the centre of Corsica (42.298899N, 9.153161E); (ii) between July and December, 2018 and January and December, 2019 from cattle in the Ponte‐Leccia slaughterhouse, which is the main active slaughterhouse in Corsica; (iii) from August to December, 2018 and 2019 (hunting season) from wild boars in the northeast of Corsica; and (iv) between March and August, 2019 from horses on farms after they had been used for horseback riding in the natural environment across Corsica (Figure 1).

For each animal, all ticks were collected and kept alive until identification and storage. Living ticks were identified at species level under a stereomicroscope using an identification key, and immediately stored at –80°C (Estrada‐Pena et al., 2014).

### 2.2 DNA extraction and polymerase chain reaction detection

Ticks were washed once in 70% ethanol for 5 min and then twice in distilled water for 5 min. Ticks were analyzed as pools consisting of 1–6 ticks of the same species, same stage, and collected from the same animal (Table 2). Individual ticks or pools of ticks were crushed in minimal essential medium containing antibiotics and fungicide, using the TissueLaser II (Qiagen, Hilden, Germany) at 30 cycles/s of 3 min. DNA extraction was performed on a QIAcube HT (Qiagen) using a QIAamp Cador Pathogen Minikit according to the manufacturer's instructions. DNA was eluted in 100 μl of buffer and stored at –80°C. Extraction was monitored by systematic spiking of each sample with MS2 bacteriophage and subsequent quantitative polymerase chain reaction (qPCR) to assess PCR‐inhibitory factors. Individual ticks or tick pools were tested using a set of qPCR assays for the detection of parapoxvirus (Kulesh et al., 2004; Nitsche et al., 2006) (Table 1).

**TABLE 1:** Primers and probes used for the detection and amplification of parapoxvirus and orthopoxvirus

Genus or species	Primer and probe	5′ → 3′ Sequence	Gene	Reference
*Pan‐Parapox virus*	Forward	TCGATGCGGTGCAGCAC	B2L	(Nitsche et al., 2006)
	Reverse	GCGGCGTATTCTTCTCGGAC		
	Probe	TGCGGTAGAAGCC		
*Pan‐Orthopox virus*	Forward	GAA CAT TTT TGG CAG AGA GAG CC	HA (J7R)	(Kulesh et al., 2004)
	Reverse	CAA CTC TTA GCC GAA GCG TAT GAG		
	Probe	CAG GCT ACC AGT TCA A		
*Pan‐Parapox virus*	Forward	GTG CGC GAA GGT GTC Kuleshov CA	ORF 011 (B2L)	(Friederichs et al., 2014)
	Reverse	ATGTGGCCGTTCTCCTCCATC		
*Pan‐Parapox virus*	Forward	CGAGCTTTAAATAGTGGAAACACAGC	ORF 032	(Friederichs et al., 2014)
	Reverse	GCACCATCATCCTGTACTTCCTC		

Reactions were performed on a 96‐well Applied Biosystems QuantStudio 3 Real‐Time PCR System using QuantiFast Pathogen. Internal and negative controls were included in each run. Samples with Ct ≥32 were considered as negative. Positive samples detected using qPCR were then analyzed by two different PCR protocols to obtain DNA fragments for sequencing (Table 1). The two PCR protocols target a 992 bp B2L gene fragment (open reading frame ORF 011) and a 1170 bp region within ORF 032. The ORF 011 (B2L) locus is a well‐known and commonly used target gene for sequence analysis and comparison of parapoxvirus DNA. Moreover, ORF 032 is highly heterogeneous and provides an excellent basis for the assessment of the relationship between and within parapoxvirus species (Friederichs et al., 2014). Positive samples were purified and sequenced using an Applied Biosystems model 3730XL (Fisher Scientific, Illkirch‐Graffenstaden, France). The newly generated sequences were aligned using X (ClustalW, Muscle, Mafft) via Mega X (Kumar et al., 2018).

### 2.3 Sequence alignment and phylogenetic analysis

For comparative analysis, additional partial B2L gene and ORF 032 sequences of other parapoxvirus were retrieved from GenBank and screened to remove short and duplicate sequences (Altschul et al., 1997). The final data set for phylogenetic analyses comprised 15 sequences for B2L, including three pseudocowpox sequences from this study, one pseudocowpox virus from cattle, one from reindeer, two from humans, four orf viruses, and four bovine popular stomatitis viruses. The final data set for phylogenetic analyses of ORF 032 comprised 24 sequences including seven pseudocowpox virus sequences from this study, one pseudocowpox virus from cattle, one from reindeer, three from humans, eight orf virus, and four bovine popular stomatitis viruses. Phylogenetic analyses were inferred using the maximum likelihood estimation method implemented in Mega X (Kumar et al., 2018). The bootstrap consensus tree was conducted with 1000 replicates.

### 2.4 Statistical analysis

The pathogens detected in pools were expressed as the percentage and minimum infection rate (maximum likelihood estimation (MLE)) method with 95% confidence intervals (CIs) based on the assumption that each PCR‐positive pool contained at least one positive tick (Sosa‐Gutierrez et al., 2016). Infection rate of DNA viruses was compared by using Fisher exact test (*p* < 0.05). The analysis was conducted using the R statistical platform (version 3.1.2) (Team, 2015).

## 3 RESULTS

### 3.1 Tick collection and morphological identification

In total, 3555 ticks were collected from 1549 different animals (687 cattle, 538 horses, 106 sheep, and 218 wild boars) (Table 2). Of these, 3490 (98%) were adult ticks and 1529 (43%) were female ticks. Overall, 1566 ticks were collected from 687 cattle from 83 different cattle‐breeding farms (Table 2). The most abundant species was *Rhipicephalus bursa* (*n* = 820; 52% of ticks collected in cattle), followed by *Hyalomma marginatum* (*n* = 441; 28%), *Hyalomma scupense* (*n* = 152; 10%), *Boophilus annulatus* (*n* = 78; 5%), *Ixodes ricinus* (*n* = 59; 4%), *Rhipicephalus sanguineus* s.l (*n* = 6; 0.4%), and *Haemaphysalis punctata* (*n* = 4; 0.3%) (Figure 1b). In total, 685 ticks were collected from 218 wild boars. The most abundant species was *Dermacantor marginatus* (*n *= 662; 96.6% of ticks collected in wild boars), followed by *I. ricinus* (*n* = 13; 2%), *R. bursa* (*n *= 9; 1.3%), and *H. marginatum* (*n* = 1; 0.1%). A total of 1285 ticks were collected from 538 horses from 21 farms. The most abundant species was *H. marginatum* (*n* = 707; 55% of ticks collected in horses), followed by *R. bursa* (*n* = 578; 45%). Thirty ticks were collected from 106 sheep. The only collected species was *R. bursa* (*n* = 30; 100%).

**TABLE 2:** Distribution of tick species by host and pools positive for parapoxvirus DNA 2018/2019

Number of pools with *n* ticks										Number of positive pools detected by real‐time Pan‐Parapoxvirus PCR				
Cattle 2019 (*n* = 456)														
Number of individual ticks or ticks per pool (*n*)	*R. bursa*	*H. marginatum*	*H. scupense*	*H. punctata*	*R. sanguineus*	*B. annulatus*	*I. ricinus*	*D. marginatus*	Total	Pool	*R. bursa*	*H. marginatum*	*H. scupense*	Total
1	60	55	30	4	3	12	6	0	170	1	1	2	2	5
2	25	25	6	0	0	2	5	0	63	2	1	3	1	5
3	14	13	6	0	1	4	3	0	41	3	2	1	0	3
4	17	10	4	0	0	0	1	0	32	4	2	1	0	3
5	12	5	2	0	0	4	0	0	23	5	0	0	0	0
6	30	16	11	0	0	5	5	0	67	6	4	2	2	8
Total	158	124	59	4	4	27	20	0	396	Total	10	9	5	24
Number of ticks	460	305	152	4	6	78	59	0	1064	MLE (95% CI)	2.25 (1.13–3.92)	3.04 (1.47–5.42)	3.42 (1.24–7.21)	2.32 (1.52–3.36)
Cattle 2018 (*n* = 241)														
1	12	25	0	0	0	0	1	0	38	1	0	2	0	2
2	7	9	0	0	0	0	1	0	17	2	2	1	0	3
3	8	14	0	0	0	0	1	0	23	3	2	3	0	5
4	15	5	0	0	0	0	0	0	20	4	0	0	0	0
5	14	5	0	0	0	0	0	0	19	5	0	0	0	0
6	30	1	0	0	0	0	0	0	31	6	2	0	0	2
Total	86	59	0	0	0	0	3	0	148	Total	6	6	0	12
Number of ticks	360	136	0	0	0	0	6	0	502	MLE (95% CI)	1.71 (0.68–3.43)	4.53 (1.83–8.97)	/	2.45 (1.32–4.07)
Cattle 2018/2019 (*n* = 687)														
1	72	80	30	4	3	12	7	0	208	1	1	4	2	7
2	32	34	6	0	0	2	6	0	80	2	3	4	1	8
3	22	27	6	0	1	4	4	0	64	3	4	4	0	8
4	32	15	4	0	0	0	1	0	52	4	2	1	0	3
5	26	10	2	0	0	4	0	0	42	5	0	0	0	0
6	60	17	11	0	0	5	5	0	98	6	6	2	2	10
Total	244	183	59	4	4	27	23	0	544	Total	16	15	5	36
Number of ticks	820	441	152	4	6	78	65	0	1566	MLE (95% CI)	2.01 (1.18–3.14)	3.5 (2.03–5.53)	3.42 (1.24–7.21)	2.36 (1.68–3.21)
Horses 2019 (*n* = 538)														
1	15	29	0	0	0	0	0	0	44	1	0	0	0	0
2	10	12	0	0	0	0	0	0	22	2	0	0	0	0
3	9	12	0	0	0	0	0	0	21	3	0	0	0	0
4	9	14	0	0	0	0	0	0	23	4	0	0	0	0
5	4	12	0	0	0	0	0	0	16	5	0	0	0	0
6	5	16	0	0	0	0	0	0	21	6	0	0	0	0
7	5	11	0	0	0	0	0	0	16	7	0	0	0	0
8	6	12	0	0	0	0	0	0	18	8	0	0	0	0
9	3	7	0	0	0	0	0	0	10	9	0	0	0	0
10	32	17	0	0	0	0	0	0	49	10	0	0	0	0
Total	98	142	0	0	0	0	0	0	240	Total	0	0	0	0
Number of ticks	578	707	0	0	0	0	0	0	1285					
Wild boars 2018/2019 (*n* = 218)														
1	4	1	0	0	0	0	1	33	39	1	0	0	0	0
2	1	0	0	0	0	0	2	25	28	2	0	0	0	0
3	1	0	0	0	0	0	0	22	23	3	0	0	0	0
4	0	0	0	0	0	0	2	25	27	4	0	0	0	0
5	0	0	0	0	0	0	0	19	19	5	0	0	0	0
6	0	0	0	0	0	0	0	53	53	6	0	0	0	0
Total	6	1	0	0	0	0	5	177	189	Total	0	0	0	0
Number of ticks	9	1	0	0	0	0	13	662	685					
Sheep 2019 (*n* = 106)														
1	30	0	0	0	0	0	0	0	30	1	0	0	0	0
Total	30	0	0	0	0	0	0	0	30	Total	0	0	0	0
Number of ticks	30	0	0	0	0	0	0	0	30					

Abbreviation: MLE, maximum likelihood estimation.

### 3.2 Detection of pathogens

Overall parapoxvirus DNA was detected in 6.6% (36/544) of tick pools collected from 23 cows from 20 farms (Table 3 and Figure 1) with an infection rate (MLE) of 2.36% (95% CI: 1.68%–3.21%). The parapoxvirus DNA detection was 8% (12/148) in 2018 and 6.0% (24/396) in 2019 (*p* = 0.57) with an MLE of 2.45% (95% CI: 1.32%–4.07%) and of 2.32% (95% CI: 1.52%–3.36%), respectively (Table 2).

**TABLE 3:** Tick species pools positive for parapoxvirus DNA

Pools ID	Tick species	Farms	Cattle ID	Number of pools per cattle	Number of cattle per Farm	Province	Sample	B2L accession number	ORF 032 accession number
2019 75	*H. marginatum*	CAL1	8520	5	1	Calcatoghju	/	/	/
2019 78	*H. marginatum*								
2019 79	*H. marginatum*								
2019 91	*H. marginatum*	LAV1	7093	7	1	Lavatoghju	PCPVCorsica2019I	MW911454	MW911458
2019 265	*H. marginatum*	OLM1	2923	4	1	Olmi‐Cappella	/	/	/
2019 268	*H. marginatum*	OLM2	1273	1	1				
2019 259	*H. marginatum*	OLM3	8821	3	1	Olmu			
2019 74	*H. marginatum*	NA1	NA4	4	/	Unknown			
2019 96	*H. marginatum*		NA5	7					
2019 26	*H. scupense*	CAS1	3186	6	1	Casanova			
2019 27	*H. scupense*								
2019 25	*H. scupense*	MOL1	4135	1	1	Moltifau			
2019 24	*H. scupense*	POP1	3256	2	1	Pulasca			
2019 22	*H. scupense*	VAL1	4607	2	1	Valle di Rustinu			
2019 306	*R. bursa*	LAV2	309	4	2	Lavatoghju			
2019 307	*R. bursa*								
2019 308	*R. bursa*								
2019 309	*R. bursa*								
2019 310	*R. bursa*		310	2					
2019 311	*R. bursa*								
2019 215	*R. bursa*	NA2	NA9	4		Unknown			
2019 217	*R. bursa*								
2019 218	*R. bursa*								
2019 272	*R. bursa*	FAR1	2018	1	3	Faringule	PCPVCorsica2019II	MW911455	MW911459
2018 2	*R. bursa*	FIL1	50	2	1	Filicetu	PCPVCorsica2018III	/	MW911462
2018 3	*H. marginatum*		50				PCPVCorsica2018I	MW911453	*MW911460*
2018 9	*R. bursa*	POR1	5		1	Portivechju	PCPVCorsica2018IV	/	MW911456
2018 10	*R. bursa*	SAN1	6825	5	1	San Martinu di Lotta	/		/
2018 12	*R. bursa*		6825				PCPVCorsica2018V		MW911457
2018 13	*H. marginatum*		6825				PCPVCorsica2018II		MW911462
2018 14	*H. marginatum*		6825				/		/
2018 15	*H. marginatum*	MON1	5687	1	1	Monticellu			
2018 101	*R. bursa*	ZIL1	6924	1	1	Zilia			
2018 102	*H. marginatum*	LENT1	8523	1	1	Lentu			
2018 103	*R. bursa*	PIE1	621	1	1	Pietralba			
2018 105	*H. marginatum*	PIE2	1823	1	2	Nessa			

The parapoxvirus DNA infection rate detected in *H. marginatum, H. scupense*, and *R. bursa* was not significantly different between these three tick species (*p* = 0.73) (Table 2). The 2018 infection rate of *R. bursa* (7%; 6/86) (MLE = 1.71% (95% CI: 0.68%–3.43%)) was similar to that observed in 2019 (6.5%; 10/154) (MLE = 2.25% (95% CI: 1.13%–3.92%)) (*p* = 1). Similar infection rates were also observed for *H. marginatum* in 2018 (10.1%, 6/59) (MLE = 4.53% (95% CI: 1.83%–8.97%)) and 2019 (7.6%, 9/124) (MLE = 3.04% (95% CI: 1.47%–5.42%)) (*p* = 0.57). *H. scupense* was collected only in 2019 (Table 2). Parapoxvirus DNA was not detected in tick pools collected from horses, wild boars, or sheep. Orthopoxvirus DNA was not identified in any of the 3555 ticks collected.

### 3.3 Phylogenetic analysis

**FIGURE 2:**
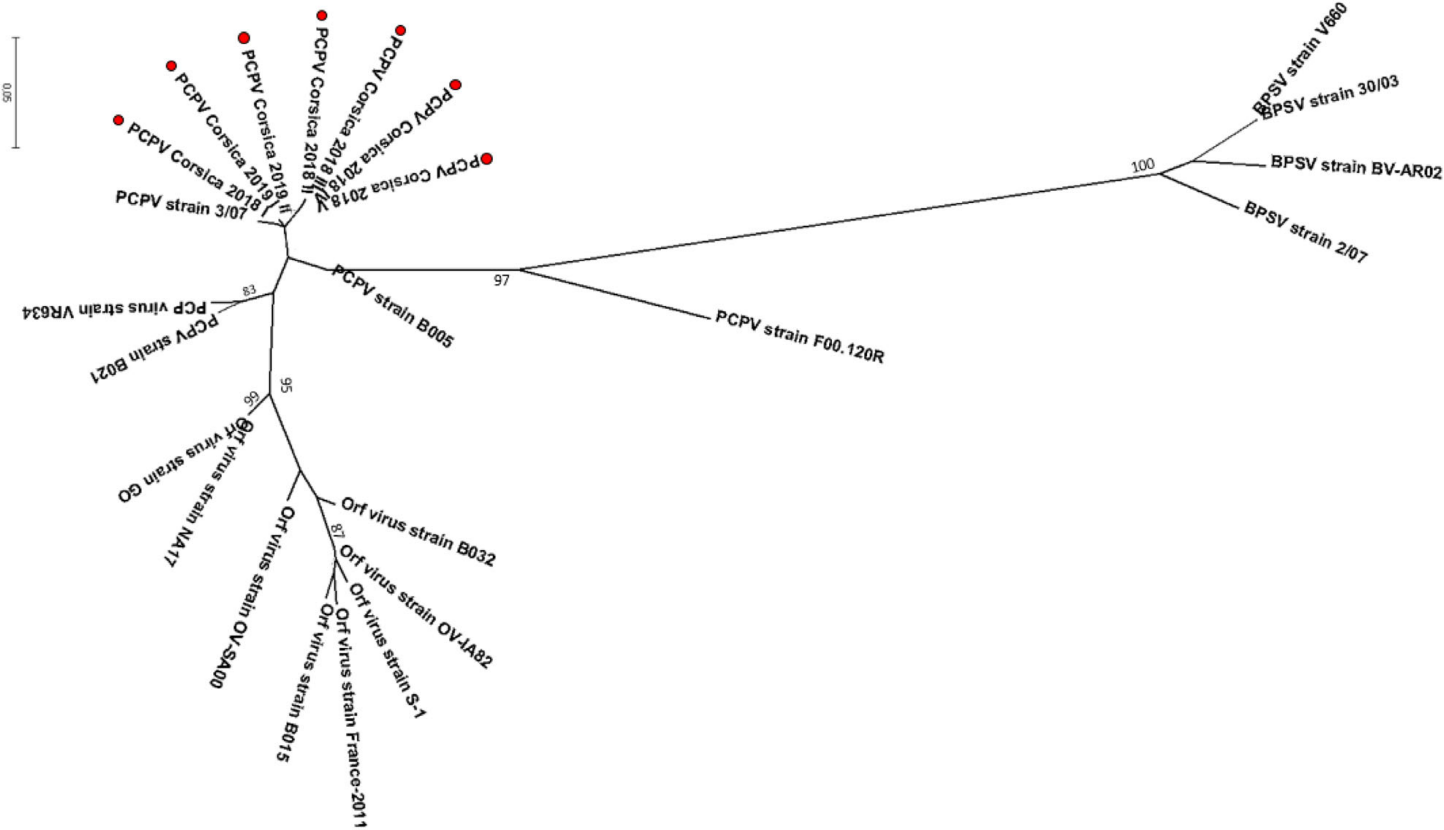
Phylogenic radiation tree of parapoxvirus‐group based deduced of 292 amino acid sequences of ORF 032 gene of parapoxvirus. The analysis was performed using a maximum‐likelihood method with JTT matrix‐based model with 1000 replicates (only values higher than 70% are shown). This analysis involved 24 amino acid sequences

**FIGURE 3:**
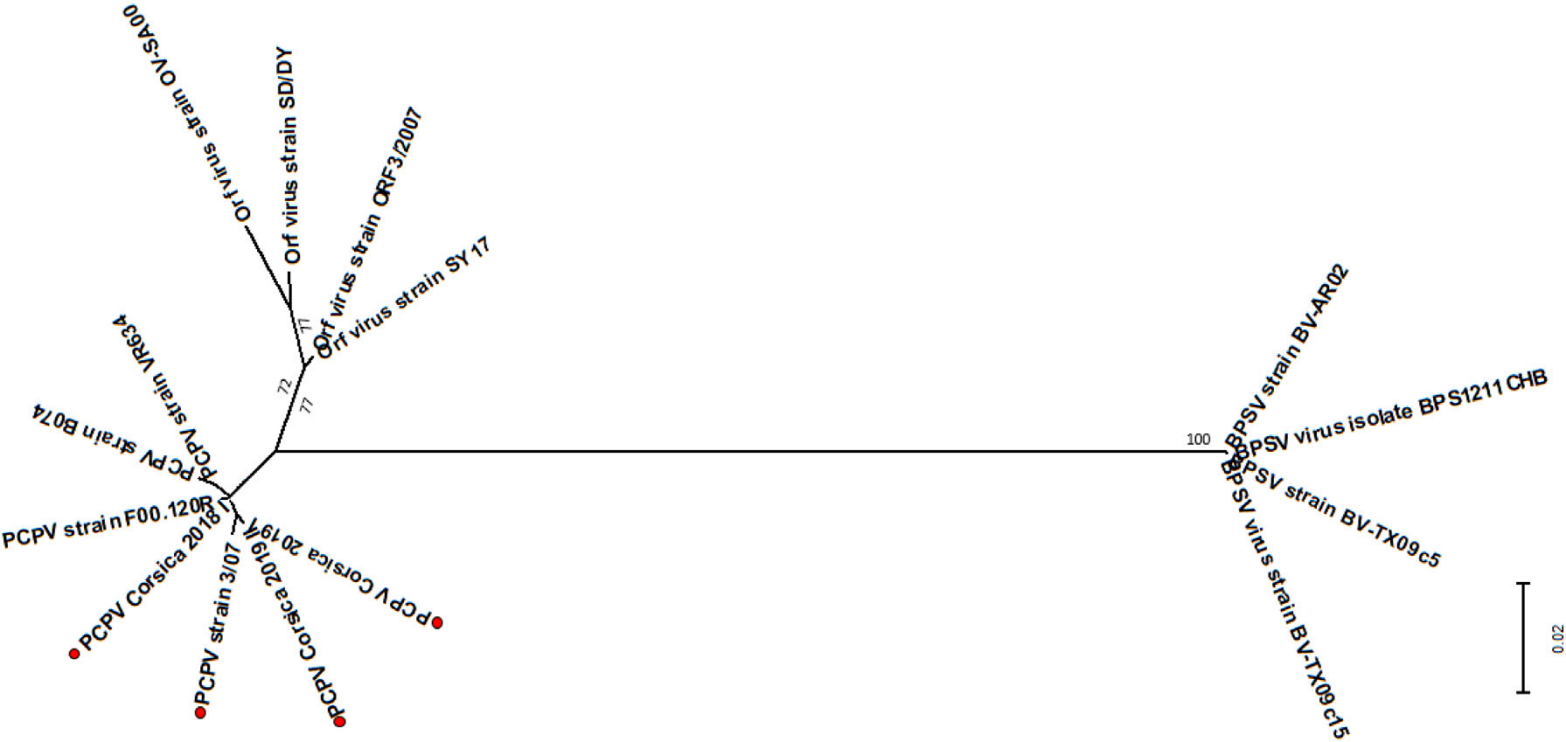
Phylogenic radiation tree of parapoxvirus‐group based deduced of 297 amino acid sequences of B2L gene of parapoxvirus. The analysis was performed using a maximum‐likelihood method with JTT matrix‐based model with 1000 replicates (only values higher than 70% are shown). This analysis involved 15 amino acid sequences

We successfully sequenced 19.4% (7/36) of the positive tick pools. The seven sequences were obtained from ticks collected from five cows belonging to seven farms (Table 3). Three B2L sequences were obtained from two *H. marginatum* pools and from one *R. bursa* pool. The phylogenetic tree based on B2L gene sequences indicated that the three samples showed 99% and 100% nucleotide and amino acid identity, respectively. The three sequences showed 99% nucleotide identity and 100% amino acid identity with parapoxvirus strain 3/07 (GenBank: KF478804) detected from cattle in Germany, with strain VR634 (GenBank: GQ329670) detected in humans in the United States and strain B074 (GenBank: KF478803) detected in humans in Germany. The seven ORF 032 gene sequences were obtained from four *R. bursa* pools and three *H. marginatum* pools. The seven sequences showed 99%–100% nucleotide and amino acid identity with each other, 98% and 99.8% nucleotide and amino acid identity, respectively, with strain 3/07 (GenBank: KF478816), and 95% and 99.5% nucleotide and amino acid identity, respectively, with strain VR634 (GenBank: GQ329670). Overall, phylogenetic tree analysis based on amino acid sequences of B2L and ORF 032 genes (Figures 2 and 3) showed that the B2L and the ORF 032 gene of parapoxvirus detected in ticks collected from cattle in Corsica were similar to each other and grouped together with pseudocowpox virus.

## 4 DISCUSSION

We report evidence of the detection of parapoxvirus DNA in three main tick species collected from cattle in Corsica. Parapoxvirus DNA was detected at similar rates in pools of *H. marginatum*, *H. scupense*, and *R. bursa* ticks, and throughout the entire 2018–2019 period of collection, showing that parapoxvirus may circulate endemically in Corsica. The results of this study showed that overall parapoxvirus DNA was detected in 6.6% of tick pools collected from 23 cows from 20 farms, demonstrating the wide circulation of poxviruses in bovine herds in Corsica. Sequence analyses showed that at least 19% of the parapoxvirus DNA detected in ticks belonged to pseudocowpox virus. In the phylogenetic reconstruction, all Corsican pseudocowpox viruses clustered with previously published European sequences of pseudocowpox viruses detected in cattle and humans. Although parapoxvirus is reportedly present in cattle worldwide (Cargnelutti et al., 2012; Ohtani et al., 2017; Ziba et al., 2020), there is no published record of the disease at the human or animal health level in Corsica. Therefore, this report marks the first identification of parapoxvirus and pseudocowpox virus in the island. The detection rate of parapoxvirus DNA in 6.6% of tick pools collected in this study was lower than the detection rate (14% parapoxvirus DNA) reported in ticks collected from cattle in Burkina Faso (Ouedraogo et al., 2020), although the percentage of positive pseudocowpox virus was similar (8.2%). No detection of parapoxvirus DNA in ticks collected from the other animal species (horses, wild boar, and sheep) and identification of parapoxvirus in different tick species suggest that ticks became infected through their blood meal from infected cattle and probably do not contribute to virus circulation. No orthopoxvirus DNA was found in ticks collected during this study in Corsica. This could be explained by the capacity for reinfection of the parapoxvirus group and the subsequent permanent circulation of that virus in the same herd, thereby inhibiting infection with the orthopoxvirus group (Mercer & Weber, 2007). However, coinfections of pseudocowpox virus and orthopoxvirus have been described in samples from lesions in cows and humans during bovine vesicular disease outbreaks in Brazil in 2015 (Abrahão et al., 2010). These two viruses have also been detected in milk from affected dairy cows (de Oliveira et al., 2018).

Finding the DNA of parapoxvirus in feeding ticks is only a marker of circulation of this genus in the cattle population; this detection cannot highlight the role of ticks in the transmission or circulation of these viruses. Implication of ticks in epidemiological cycle of parapoxvirus should be tested in laboratory through vector competence studies to have a comprehensive idea of their real implication. Moreover, we have no data on the impact on animal health of parapoxvirus positive tick hosts. Working with pooled ticks has several advantages but inevitably poses problems with prevalence estimates. Seven of the 36 positive samples were able to be sequenced and analysis showed the presence of pseudocowpox virus. Hence, it is possible that other viruses of the genus were present.

In conclusion, this study showed that parapoxvirus circulates in cattle in Corsica. Therefore, a broad surveillance is crucial to provide data that elucidate the origin and dissemination dynamics of parapoxvirus to investigate the prevalence of parapoxvirus infections in the cattle population and identify infection risks for other animals and humans.

## CONFLICT OF INTEREST

The authors declare no conflict of interests.

## AUTHOR CONTRIBUTIONS

Cicculli Vincent, Ayhan Nazli, and Falchi Alessandra conceived the study, analyzed data, and drafted the manuscript. Cicculli Vincent, Pezzi Laura, Luciani Léa, Decarreaux Dorine, and Maitre Apolline were involved in microbiological diagnosis. Decarreaux Dorine, Maitre Apolline, and Cicculli Vincent collected ticks. N. de Lamballerie Xavier, Vial Laurence, Paoli Jean‐Christophe, and Charrel Remi drafted the manuscript.

## ETHICS STATEMENT

No ethical approval was required, as this study does not involve clinical trials or experimental procedures. The cattle inspected were slaughtered for human consumption. Living sheep and Horses were examined with the assistance of their owner. This study did not involve endangered or protected species. The wild boars collected were legally hunted during the hunting season.

### PEER REVIEW

The peer review history for this article is available at https://publons.com/publon/10.1002/vms3.700.

## Data Availability

The data that support the findings of this study are available from the corresponding author upon reasonable request.
